# Prevalence of Work-Related Musculoskeletal Disorders Among Orthopedic Surgeons in Saudi Arabia: A Systematic Literature Review

**DOI:** 10.7759/cureus.72634

**Published:** 2024-10-29

**Authors:** Omar A Bokhary, Mohammad A Alsolami, Anmar K Alkindy, Mahmod S Numan, Mohammed A Jumah, Amro A Mirza

**Affiliations:** 1 Department of Orthopaedic Surgery, King Abdulaziz University Hospital, Jeddah, SAU; 2 Department of Orthopaedic Surgery, Dr. Soliman Fakeeh Hospital, Jeddah, SAU; 3 Department of Orthopaedic Surgery, King Abdulaziz Medical City, Ministry of National Guard Health Affairs, Jeddah, SAU; 4 Faculty of Medicine, King Abdulaziz University, Jeddah, SAU; 5 Department of Family Medicine, Ministry of Health, Jeddah, SAU; 6 Department of Orthopaedic Surgery, King Fahad General Hospital, Jeddah, SAU

**Keywords:** back pain, musculoskeletal disorders, review, saudi arabia, work-related

## Abstract

Work-related musculoskeletal disorder (WRMD) complications are common in surgeons in general, but they are much higher in orthopedic surgeons. Numerous studies have explored the prevalence of WRMD among orthopedic surgeons internationally. However, little research has been carried out in Saudi Arabia on this matter. This review aimed to investigate it. A consistent and systematic search method was carried out to establish studies reporting the prevalence of WRMD among orthopedic surgeons in Saudi Arabia. The search comprised published research from 2000 to June 2024. Nine eligible studies were incorporated for analysis, which involved 490 participants. The prevalence documented in these studies has exhibited an upward trend in recent years, with reported rates ranging from 36% to 90.3%. The most frequent anatomic site is found to be the lower back. There is a significant association between WRMD, smoking, and increasing age. The prevalence of WRMD is elevated among orthopedics based on existing information in the literature. Hospitals, along with orthopedic residency programs offering orthopedic-specialized ergonomics or occupational injury lectures as well as workshops, may help equip surgeons with the necessary knowledge and skills for addressing this issue.

## Introduction and background

Work-related musculoskeletal disorders (WRMDs) are challenging and are becoming a more common problem in workplaces in our society. The World Health Organization (WHO) has stated that 37% of back pain worldwide is caused by various occupational settings [1]. Physicians are liable to bowing, twisting, and maintaining an uncoordinated position for a long period, which are all known to be risk components for WRMD [2]. WRMD complications and incidence are common in surgeons in general, but they are much higher in orthopedic surgeons. It mostly affects the upper extremity, neck, and back regions [3]. Orthopedic surgery is a physically and mentally demanding specialty; thus, orthopedic surgeons are more prone to WRMD than their contemporaries. The pain and discomfort experienced are frequently caused by various contributory variables that all connect to either the lengthy and physically rigorous operations or likely the unconventional postures that orthopedics are put into throughout the surgeries [4,5]. The prevalence is the measure of current cases within a population at the time of a particular research period. Previous studies conducted in the United States on this topic reported an increased prevalence of WRMD among orthopedic surgeons, with some cases requiring surgical intervention [3,6]. Furthermore, a study conducted in Saudi Arabia comparing the prevalence of WRMD between surgical and medical specialty residents found that being a surgeon and spending time doing interventional procedures are predisposing factors for musculoskeletal pain [2]. Multiple studies have attempted to study WRMDs' prevalence and anatomic sites in orthopedic surgeons [2,3,6]. However, research on this topic is very little in Saudi Arabia. This review aims to investigate WRMD prevalence among orthopedics and its associations in Saudi Arabia.

## Review

Materials and methods

This review conformed to the criteria defined in the Preferred Reporting Items for Systematic Reviews and Meta-Analyses (PRISMA). The authors conducted electronic searches on PubMed and Google Scholar to systematically identify relevant published studies from 2000 to June 2024. The search terms included "Work-Related Musculoskeletal Disorders in Orthopedic Surgeons", "prevalence or incidence or epidemiology", and "Saudi Arabia". The search words were connected in varied ways to find applicable literature, and the search methods were modified for respective databases. In addition, relevant studies were sought in the reference lists of eligible articles.

The review included studies that focused specifically on orthopedic surgeons in Saudi Arabia and reported statistics on the prevalence of WRMDs, categorized by age, sex, and anatomical site. Eligible studies employed either cluster sampling or random sampling methods and were published between the year 2000 and June 2024.

Studies conducted outside Saudi Arabia and studies published prior to the year 2000 were excluded. Additionally, studies where the Digital Object Identifier (DOI) is not functional or the article is otherwise inaccessible through available academic databases or direct contact with the journal, studies that did not explicitly identify orthopedic surgeons within their sample, those that focused on specialties other than orthopedics, and studies addressing work-related disorders other than musculoskeletal conditions were also excluded.

The reviewers individually appraised every article's title and abstract based on the inclusion and exclusion criteria. Studies that did not meet the inclusion criteria were excluded. To prevent doubt throughout all screening stages, we conducted further analysis and addressed issues through discussion to reach a consensus. Studies in the complete text were assessed for acceptability. The review comprised the remaining studies.

The reviewers extracted the data, which included the following: (1) first author, (2) study year, (3) study design, (4) average age, (5) sex, (6) rate of WRMD, (7) anatomic site of WRMD, (8) association with age, (9) association with sex, (10) association with smoking, and (11) association with residency year.

Results

**Figure 1:**
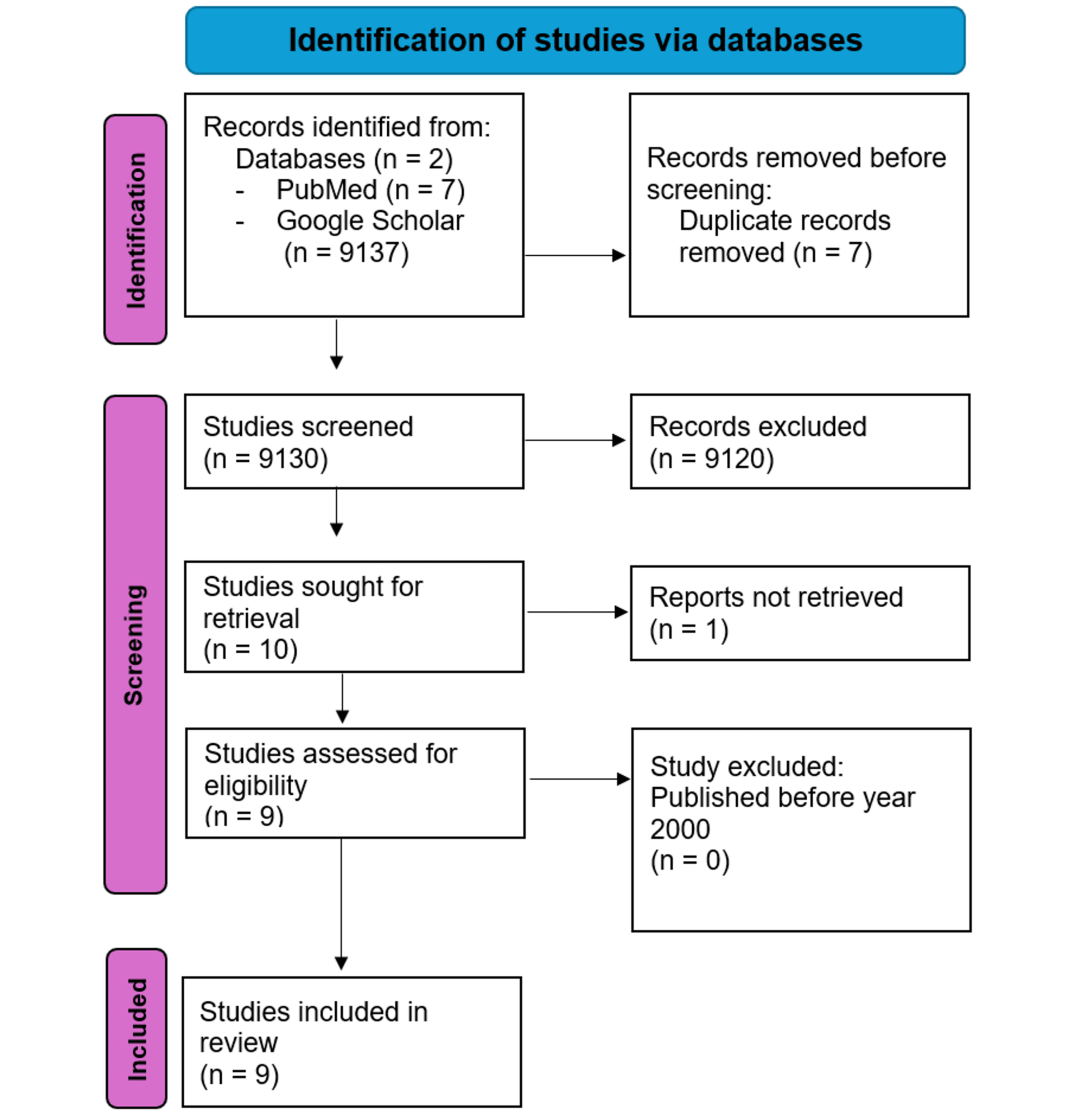
Search and screening flowchart.

The systematic database search provided articles; after discarding duplicates, 9130 articles were reviewed by titles/abstracts, and 9121 were discarded. Nine of these articles were then reviewed in complete text, yielding nine included publications (Figure 1).

Table 1 shows the characteristics of the included studies.

**Table 1:** Characteristics of the included studies. *Only this anatomic site was investigated. WRMD: work-related musculoskeletal disorder

Author	Year	Type of study	City (region)	Total participants	Mean age	Rate of WRMD	Males N (%)	Females N (%)	Most common anatomic site of WRMD	Association with age	Association with gender	Association with smoking	Association with residency year
AlHussain et al. [7]	2023	Cross-sectional	Riyadh (Central)	50	-	Occasional occurrences (36%)	44 (88%)	6 (12%)	Shoulder^*^	20-30: 0.037, 31-40: 0.087, 41-50: 0.029	-	-	-
Alshareef et al. [8]	2023	Cross-sectional	All regions	17	-	14 (82.3%)	-	-	Back and neck pain*	-	-	-	-
Al Mulhim et al. [9]	2023	Cross-sectional	Eastern region	83	27.8 ± 2.2	63 (75.9%)	52 (62.7%)	31 (37.3%)	Lower back pain	0.976	0.803	0.043	0.049
Aseri et al.[10]	2019	Cross-sectional	Jeddah (Western)	25	-	14 (56%)	-	-	Low back pain*	-	-	-	-
Al-Ruwaili and Khalil [11]	2019	Cross-sectional	Tabuk (Northern)	15	-	9 (60%)	-	-	Low back pain*	-	-	-	-
Alzidani et al.[12]	2018	Cross-sectional	Taif (Western)	10	-	8 (90.3%)	-	-	Low back pain*	-	-	0.033	-
Aljohani et al.[13]	2020	Cross-Sectional	Almadinah Almunawwarah, Tabuk	97	-	49.50%	90 (93%)	7	Lower back pain	-	-	-	-
Alnefaie et al. [14]	2019	Cross-sectional	Jeddah (Western)	14	-	8 (57.1%)	-	-	-	-	-	-	-
Al-Mohrej et al. [15]	2020	Cross-sectional	Riyadh (Central)	179	32.2 ± 7.7	67%	173 (96.6%)	6 (3.4%)	Lower back pain	0.247	0.945	0.38	-

A total of nine studies were incorporated into this review (Table 1), all of which utilized a cross-sectional design. Of these, four studies focused specifically on orthopedic surgeons [7,9,13,15], while the remaining five included multiple surgical specialties, including orthopedics. The reported prevalence of WRMDs varied across studies, with the highest rates being 90.3% and 75.9%, as observed by Alzidani et al. and Al Mulhim et al., respectively [9,12]. Conversely, the lowest reported prevalence rates were 36% and 49.5%, as found by AlHussain et al. and Aljohani et al. [7,13]. In terms of the anatomical distribution of WRMD, three studies that examined multiple anatomical sites concluded that lower back pain was the most prevalent musculoskeletal complaint among participants [9,13,15]. Additionally, four studies limited their focus specifically to lower back pain [8,10-12]. And one only investigated shoulder pain [7]. Another study did not specify which anatomical site was most affected within the orthopedic surgeon population [14].

Al Mulhim et al. (P=0.043) and Alzidani et al. (P=0.033) reported significant findings from their studies that examined the relationship between smoking and WRMD [9,12]. Al-Mohrej et al. found no significant association [15]. Two studies looked into WRMD in terms of gender; however, neither discovered a statistically significant relationship [9,15]. Only one study examined WRMD in relation to residency year and found a significant association (P=0.049) [9]. Finally, three studies investigated age as a variable [7,9,15]; only one of them found a significant connection (P=0.029) [7].

Discussion

Studies included in the review showed that there is a high rate of WRMD among orthopedic surgeons in Saudi Arabia [7-15], ranging from 36% up to 90.3%. These findings are consistent with a study conducted in 2022 by the American Academy of Orthopaedic Surgeons (AAOS) [6], with 86% of surgeons reporting a minimum of one musculoskeletal condition. Another study conducted by the Orthopedic Korean Society of Spine Surgery showed that 78.8% of the participants experienced WRMD in the past year [16]. A recent review done by Vasireddi et al. on the prevalence of WRMD in orthopedics and the ergonomics involved in surgery concluded that there is a high career prevalence of WRMD in orthopedics, as well as very minimal efforts to enhance surgical ergonomics [17]. According to Epstein et al., many surgical training residency programs do not give official (98%) or unofficial (75%) training for surgical ergonomics [18]. It suggests that physicians, even orthopedic surgeons, may not have the availability to training on evidence-based ergonomics of surgery, increasing their liability of developing WRMD later during their future careers. Institutions and orthopedic surgery residency training should provide orthopedic-specialized ergonomics or lectures on work injury to help educate surgeons on this specific issue.

The most common anatomic site for WRMD among the studies that investigated all sites in the review is lower back pain [9,13,15]. This is in accordance with a study published on orthopedic residents at the University of Iowa in 2014, which concluded a 55% lower back pain prevalence among the participants, the second highest in their sample [3]. Furthermore, another study conducted on orthopedic surgeons in New York concluded a 77% prevalence of back pain among respondents [19]. The rate of musculoskeletal pain, particularly back pain, is very high among orthopedic surgeons. This could be explained firstly by the prolonged durations of orthopedic surgeries which could reach to more than 10 hours depending on the case complexity and sub-specialty of the surgeon. Secondly, manipulating limb and application of force is an important part of the surgeries, and the lower back plays a crucial role in maintaining balance and supporting these movements. 

Two studies included in the review found a significant association between smoking and WRMD [9,12]. It is well-documented that smoking is associated with regional pain [20]. Furthermore, previous studies investigating the effect of smoking on muscular and bone pain have collectively shown that it adversely affects bone mineral density and increases the number of fractures and bone and wound curing [21,22]. Thus, a significant association is logical. We recommend enhancing awareness among orthopedic surgeons, as their specialized surgical practices place them at heightened risk for developing musculoskeletal disorders. As previously noted, smoking exacerbates these risks, further compromising their health and professional performance.

Our review's limitations include a limited number of articles published regarding WRMD among orthopedic surgeons in the country and six studies only investigating one anatomic site. However, we demonstrated a pattern of possible elevated prevalence among orthopedic surgeons, which is crucial for the healthcare system.

## Conclusions

Studies on WRMD among orthopedic surgeons are limited in Saudi Arabia. Despite this, an elevated prevalence of WRMD ranging from 36% to 90.3% was concluded. The lower back is the most frequent anatomic site among the studies, with variables such as smoking and increasing age having a significant association. We suggest that future studies explore the impact of patient and surgeon positions in the operating room and their association with musculoskeletal disorders to better understand their causes and increase awareness. Hospitals and orthopedic surgery residency programs should prioritize offering orthopedic-specialized ergonomics or work injury workshops to equip surgeons with the necessary knowledge and skills for addressing this critical issue.
